# What Global Oncology Needs: Mutual Learning and More Funding

**DOI:** 10.1200/JGO.17.00237

**Published:** 2018-02-08

**Authors:** Bishal Gyawali, Gilberto Lopes

**Affiliations:** **Bishal Gyawali**, Anticancer Fund, Belgium, and Civil Service Hospital, Kathmandu, Nepal; and **Gilberto Lopes**, University of Miami, Miami, FL

Although we have not yet agreed to an official definition of global oncology, one of the overarching themes of this emerging medical discipline is cancer control in low- and middle-income countries (LMICs), given that 65% of cancer deaths worldwide occur in these countries.^1^ Hence, to control the global cancer burden, it is important to address the burden of cancer in LMICs. However, it is also important to note that global oncology is not the concern of LMICs alone. Cancer respects no boundaries and neither should cancer control efforts.

The article by Kostelecky et al^2^ accompanying this editorial highlights an important pillar of global oncology—partnerships between high-income countries (HICs) and LMICs to improve cancer care and research in LMICs. In the article by Kostelecky et al^2^, the authors share important details, including the challenges and benefits of collaboration between the National Cancer Institute Center for Global Health (NCI-CGH) and Latin American countries that should inspire and encourage other partners to invest and work in other LMICs to improve global standards for cancer care. We would like to highlight some important takeaways from this article for future HIC-LMIC collaborations.

First, nobody can do it alone. The article underlines important collaborative efforts between the NCI-CGH and other organizations such as the Union for International Cancer Control, the International Agency for Research on Cancer, and other NCI and National Institutes of Health working for cancer control in the Latin American region. However, we must foster continuing contact and improve coordination among the various players, because disarrayed and disorganized efforts often lead to waste of funds without substantial improvement in outcomes. It is important that governments in LMICs take a greater share of the initiatives and ensure that the efforts from all organizations are in concert, with no duplication of efforts or squandering of resources.

Second, all cancer control efforts begin with a proper registry. Without reliable data, the actual burden of cancer, as well as the demographic details of cancer types, remains unknown and, thus, intervention efforts can be misguided and wasteful. It was pleasing to see that one of the important priorities in this NCI-CGH collaboration was the development of population-based cancer registries. It might be prudent for governments and other organizations that work in other LMICs to make the development of a reliable population-based cancer registries one of their top priorities.

Third, another important activity fostered by the NCI-CGH was the conduct of summer schools, workshops, and conferences. It is still an open question, however, if these investments actually lead to better outcomes. Although almost all participants from LMICs had positive things to say about the programs they attended, these numbers might be inflated because of deference and the fact that it is unlikely that participants who receive support to attend a training opportunity would answer negatively in such assessments. We need to create measures and performance indicators that reflect positive changes and improvements in cancer control efforts. Moreover, the creation of longer-term relationships and idea exchange would likely lead to more effective mentor-mentee relationships and could provide continued motivation and support to translate the acquired knowledge into action or practice. We believe that although such workshops and meetings are important, longer-term support is essential for visible changes in cancer care outcomes. For example, would supporting two colleagues from LMICs to attend training in pathology, surgery, radiology, or chemotherapy for 6 months provide better outcomes compared with supporting 50 participants to attend a 7-day course at similar costs? It would be interesting if these strategies were tested as part of a trial to inform evidence-based policy investment.

Last, but most importantly, global oncology efforts should not be seen as a one-sided pedagogy. HICs have as much to learn and gain from LMICs by working together. LMICs have slowly but surely understood the importance of investing in clinical research and some high-quality cancer trials of global importance originated in LMICs.^3,4^ It is interesting to note that although trials conducted in LMICs are, on average, of lower quality than trials conducted in HICs, trials conducted in both LMICs and HICs tend to be of an even higher quality than trials done only in HICs.^5^ Thus, future collaborations should focus more on teaching how to fish rather than providing fish (ie, help LMICs build capacities for clinical research and trials to innovate and implement ideas of local importance rather than to simply encourage LMICs to copy what HICs do).^6^ History is replete with examples of HICs collaborating with LMICs to co-develop low-cost and effective interventions adapted to the needs of LMICs.^7,8^

On the other hand, one of the perils of such efforts from international organizations for cancer control in LMICs that deserves due consideration is the abdication of responsibilities by local governments. LMICs should take proactive steps on their own and list evidence-based priorities for interventions that are essential to their local population. For example, although many LMICs have begun to incorporate mammography screening into their national health agenda, the cost-effectiveness and the utility or need of this intervention to LMICs has not been properly studied.^9^ Having evidence-based priorities set and stratified by cost-effectiveness would enable better coordination between international and local efforts so we can meet such objectives without squandering resources into areas in which the need is not as dire or urgent.

We would like to end by congratulating Kostelecky et al^2^ for sharing these otherwise behind-the-door activities in global oncology. Their article is a nice reminder of the important, but not always publicized, direct and indirect benefits of funding organizations such as NCI-CGH for their work in LMICs. Threats of funding cuts would only hinder the already meager, tentative steps we have started to take toward global cancer control.^10,11^ Funding cuts are also demoralizing to the researchers who devote themselves to the noble pursuit of global cancer control. Unlike “cancer moonshots,” global cancer control, or what we prefer to call “cancer groundshot” projects, do not look attractive enough to gather substantial funding from private organizations. Thus, it is imperative that the public organizations working on global health and global oncology receive enough uninterrupted funds. Global oncology needs more funding, not less.
